# Population Structure of Humpback Whales from Their Breeding Grounds in the South Atlantic and Indian Oceans

**DOI:** 10.1371/journal.pone.0007318

**Published:** 2009-10-08

**Authors:** Howard C. Rosenbaum, Cristina Pomilla, Martin Mendez, Matthew S. Leslie, Peter B. Best, Ken P. Findlay, Gianna Minton, Peter J. Ersts, Timothy Collins, Marcia H. Engel, Sandro L. Bonatto, Deon P. G. H. Kotze, Mike Meÿer, Jaco Barendse, Meredith Thornton, Yvette Razafindrakoto, Solange Ngouessono, Michel Vely, Jeremy Kiszka

**Affiliations:** 1 Cetacean Conservation and Research Program, Global Conservation-Marine, Wildlife Conservation Society, Bronx, New York, United States of America; 2 Sackler Institute for Comparative Genomics and Conservation Genetics Program, American Museum of Natural History, New York, New York, United States of America; 3 American Museum of Natural History, Center for Biodiversity and Conservation, New York, New York, United States of America; 4 Department of E3B, Columbia University, New York, New York, United States of America; 5 University of Pretoria, Mammal Research Institute, Pretoria, Cape Town, South Africa; 6 Environment Society of Oman, Ruwi, Muscat, Sultanate of Oman; 7 Instituto Baleia Jubarte, Caravelas, Bahia, Brazil; 8 Oceanography Department, University of Cape Town, Rondebosch, South Africa; 9 Marine and Coastal Management, Rogge Bay, South Africa; 10 Faculdade de Biociências, Pontifícia Universidade Católica do Rio Grande do Sul, Porto Alegre, Rio Grande do Sul, Brazil; 11 Wildlife Conservation Society-Madagascar Country Program, Antananarivo, Madagascar; 12 Agence Nationale des Parcs Nationaux, Ministère du Tourisme et des Parcs Nationaux, Libreville, Gabon; 13 Association Megaptera, Paris, France; 14 Université de La Rochelle, LIENSS, Institut du Littoral et de l'Environnement, La Rochelle, France; American Museum of Natural History, United States of America

## Abstract

Although humpback whales are among the best-studied of the large whales, population boundaries in the Southern Hemisphere (SH) have remained largely untested. We assess population structure of SH humpback whales using 1,527 samples collected from whales at fourteen sampling sites within the Southwestern and Southeastern Atlantic, the Southwestern Indian Ocean, and Northern Indian Ocean (Breeding Stocks A, B, C and X, respectively). Evaluation of mtDNA population structure and migration rates was carried out under different statistical frameworks. Using all genetic evidence, the results suggest significant degrees of population structure between all ocean basins, with the Southwestern and Northern Indian Ocean most differentiated from each other. Effective migration rates were highest between the Southeastern Atlantic and the Southwestern Indian Ocean, followed by rates within the Southeastern Atlantic, and the lowest between the Southwestern and Northern Indian Ocean. At finer scales, very low gene flow was detected between the two neighbouring sub-regions in the Southeastern Atlantic, compared to high gene flow for whales within the Southwestern Indian Ocean. Our genetic results support the current management designations proposed by the International Whaling Commission of Breeding Stocks A, B, C, and X as four strongly structured populations. The population structure patterns found in this study are likely to have been influenced by a combination of long-term maternally directed fidelity of migratory destinations, along with other ecological and oceanographic features in the region.

## Introduction

The issue of population structure has remained a central one in the areas of molecular ecology and conservation biology. An understanding of ecological forces influencing dispersal and isolation by applying robust and high-resolution molecular tools has aided in conservation management planning [1]. A recurrent topic in the marine environment centers on the varying degrees and plausible mechanisms of population structure for different species. In marine systems, barriers to gene flow are often not as conspicuous as they are in terrestrial environments [2], making it difficult to interpret gene flow and population differentiation patterns [3]. For example, inter and intra-specific differences between Atlantic and Pacific oceans are thought to have emerged by the formation of the Panama Land Bridge, ice barriers in the Arctic, or by differential environmental tolerance through the Arctic or the tropics [4], [5]. Conversely, periods of inter-glacial warming have allowed episodic contact between populations in the Southern and Northern Hemispheres over evolutionary time scales [6]. Barriers to dispersal have not fluctuated to the same extent throughout large parts of the Southern Hemisphere (SH) [7], and therefore populations of large migratory marine species in the SH may potentially show a higher degree of connectivity across ocean basins over evolutionary and demographic timeframes [8], [9].

Social structure, long-term maternally reinforced migratory behaviour, and vicariant events can have profound effects on the degree of population structure detected among marine species [10]. Ecological and environmental discontinuities such as frontal systems, ocean currents, and abrupt changes in bathymetry can also function as marine boundaries for some marine species [11], [12], [13]. Despite the plausibility of these mechanisms affecting population structure, we often lack sufficient information to empirically test the significance of such relationships. An exception to this general lack of information is the case of sea turtles [14], which exemplifies the influence of philopatry on phylogeographical patterns, and in maintaining significant and very high levels of genetic differentiation (Fst∼0.5), despite transoceanic movements and oceanographic influences [9], [15]. Some species of large migratory whales in the SH offer an opportunity to examine population differences at large oceanic scales and evaluate the influence of population history [16], [17], but the influence of ecological phenomena and environmental features on such population structure was not fully considered. Humpback whales are one such species to evaluate the role of ecological and evolutionary forces in shaping population structure. They are widely distributed throughout the SH, undertake long-distance migrations between feeding areas and breeding grounds, and yet typically show preference for specific coastal regions proximate to continental shelf areas or oceanic islands [18].

Much of what we know about population differentiation among humpback whales has resulted from studies in the North Atlantic and North Pacific [19], [20], [21], [22], [23], [24], [25], [26]. In general, humpback whales within an ocean basin in the Northern Hemisphere (NH) mix on common sub-areas in breeding grounds but segregate and show maternal fidelity to particular sectors on their feeding areas [25], [27], [28]. This is reflected in the very high and significant fixation indices (*F_ST_*∼0.4) between feeding areas and breeding grounds in the North Pacific, and in significant indices (*K_ST_*∼0.04) between feeding areas in the North Atlantic [29], [30]. High and significant degrees of population structure (*F_ST_*∼0.1–0.28) among breeding concentrations have been detected in the North Pacific, reflecting long-term population isolation between continental and oceanic grounds proximate to Western Mexico and the Hawaiian Archipelago, respectively [19], [21], [29].

**Figure 1:**
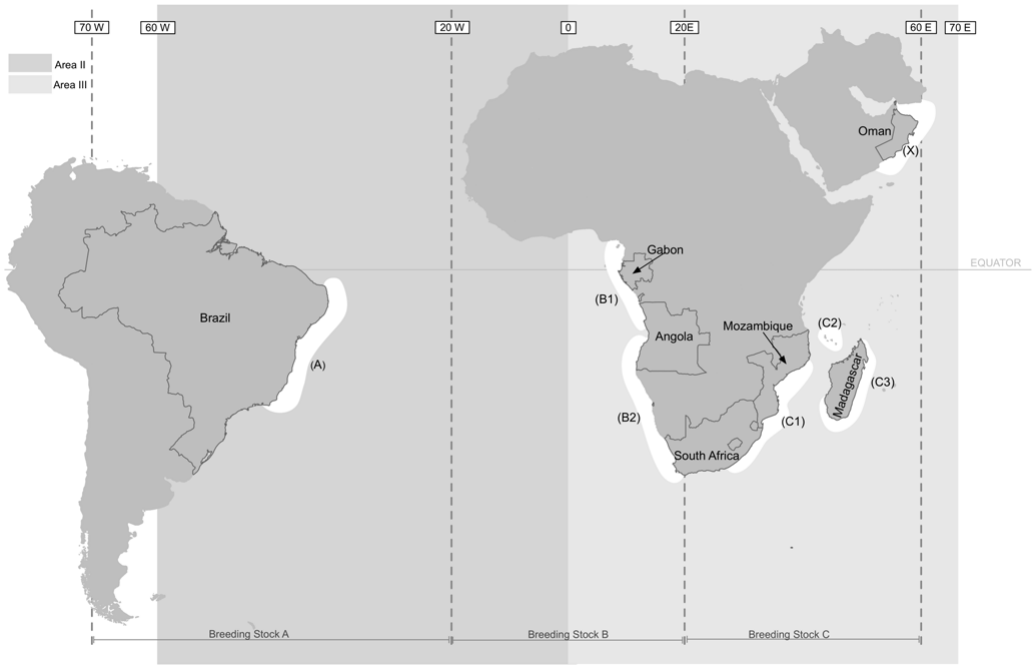
IWC boundaries for humpback whale breeding grounds and feeding areas in the South Atlantic and Indian Oceans. Sampling locations are indicated in parentheses and referred to in Table 1.

There are some suggestions that the population structure observed in the NH should apply to humpback whales in the SH but with migration polarities seasonally reversed [31]. However, other factors suggest this might not be the case, including the differences between NH and SH humpback whale migration patterns, the nature of the circumpolar Antarctic ecosystem, as well as potential differences in other biological factors (e.g. diet and metabolic rates). In the Southern Oceans, humpback whale distribution on feeding areas was historically divided into six longitudinal sectors surrounding the South Pole, termed Areas I –VI that have been subsequently used for sub-population identity [18], [32] by the International Whaling Commission (IWC). Seasonal distribution data between these high-latitude feeding areas and low-latitude breeding grounds led to the designation of seven breeding grounds and migratory corridors termed Breeding Stocks A to G [33]. An eighth Breeding and Feeding Stock (X) in the northern Indian Ocean, with no access to high-latitude waters in the NH for summer feeding, has been proposed by the IWC [34], [35] (See Fig. 1).

Further stock subdivision at the intra-oceanic spatial scale in the SH has been discussed by the IWC. Within the Southeast Atlantic, humpback whales were further characterized into B1 and B2 sub populations to the north and south of 18° S, respectively - in the vicinity of the northern end of the Walvis Ridge and the Angola-Benguela front [33], [36]. In the Southwest Indian Ocean, three separate migratory streams of the Stock C have been proposed [37], based largely on distributional evidence and catch histories; one migration and breeding stock along the continental east coast of Africa (termed C1 by the IWC), a second utilizing the waters of the central Mozambique Channel Islands (C2), and a third that travels along the south and east coast of Madagascar to the relatively sheltered Antongil Bay and potentially to other areas surrounding the coasts of Madagascar during the winter months (C3) [38], [39], [40] (See Fig. 1). The extent to which these management designations in areas of the South Atlantic and Indian Ocean reflect whale population history and population structure has never previously been tested using genetic data.

Historically, research extending through first half of the 20^th^ century used Discovery Mark Programme data from the 1930s–1960s to reveal the first clues to population structure and migratory connections between feeding areas and breeding grounds [18], . The program was established to understand population connectivity and population dynamics to better manage whaling. In some areas such as the eastern Indian Ocean (Breeding Stock D) and the Southwestern Pacific (Breeding Stock E) there were sufficient migratory connections to make inferences regarding population structure, before genetic or photographic data became available [42], [43], [47], [48]. However, in the South Atlantic and Southwestern Indian Ocean, connectivity patterns have not been established to the same extent, primarily due to fewer returns from the Discovery Mark Programme [46]. More recently, the development of individual identification, satellite telemetry and particularly genetic methodologies have provided powerful additional means of investigating cetacean population structure and even testing the validity of some potential biological and ecological boundaries of the management units used by the IWC [33].

In a first step toward this objective for SH humpback whales, Baker et al. [29] and Olavarria et al. [49] evaluated genetic data using fixation indices to assess IWC stock designations largely from the South Pacific breeding grounds (Breeding Stocks D, E, F and G). The approach provided sufficient resolution and statistical power to corroborate the large-scale oceanic stock boundaries previously proposed by the IWC.

Our study uses a multi-year and large-scale genetic sampling approach in the Indian and South Atlantic Oceans to study inter- and intra-oceanic SH humpback whale population structure. Long-term collaborative studies of humpback whales in the Southwestern Atlantic Ocean (Breeding Stock A), the Southeastern Atlantic (Breeding Stock B), the Southwestern Indian Ocean (Breeding Stock C), and the Northern Indian Ocean (Stock X) have facilitated these studies. Specifically, we evaluate (i) the degree of gene flow and mtDNA structure that exists among oceanic breeding grounds (e.g. eastern South Atlantic Ocean or Breeding Stock B, compared to Breeding Stock C in the Western Indian Ocean), (ii) the degree of gene flow and mtDNA structure among sub-populations within these oceanic breeding grounds (iii) the potential for sex-biased dispersal in relation to population structure in the breeding grounds, and (iv) the implications of population genetic structure for conservation management of this species.

Overall, our results help elucidate how ecological and evolutionary process have shaped population structure and gene flow patterns among humpback whales distributed within the South Atlantic and Indian Ocean (both North and South), and allow us to test the applicability of current stock definitions used for conservation and management of this species.

## Methods

### Sample collection and DNA sequencing

Samples representing 1,527 individual whales were collected from humpback whales across fourteen sampling sites within the South Atlantic (Stocks A and B), the Southwestern Indian Ocean (Stock C), and Northern Indian Ocean (Stock X) (Table 1, Figure 1). All research undertaken followed local regulations and guidelines for such research. This project was also approved by the American Museum of Natural History Institutional Animal Care and Use Committee (IACUC).

**Table 1:** Sample location, size, mtDNA control region variability for breeding grounds and migratory corridors of Southern Hemisphere humpback whales.

Breeding Ground Breeding Stock	Sample Size	Males	Females	Number of haplotypes	Polym. sites	h (SD)	π (SD)	Collection years
**(A) Southwestern Atlantic Ocean**								
Abrolhos, Brazil	164	20	18	66	64	0.9744 (0.0041)	0.0250 (0.0127)	1997–2001
**(B) Southeastern Atlantic Ocean**								
(B1) Gabon, Cabinda	477	321	156	100	72	0.9795 (0.0017)	0.0210 (0.0106)	1998–2002
(B2) West South Africa	108	49	55	52	60	0.9766 (0.0058)	0.0203 (0.0103)	1993–2004
**(C) Southwestern Indian Ocean**								
(C1) Mozambique & East South Africa	151	83	66	65	61	0.9790 (0.0038)	0.0195 (0.0099)	1991, 1999–2003
(C2) Mayotte & Geyser, Comoros	78	18	57	32	55	0.9740 (0.0062)	0.0214 (0.0109)	1997–2003
(C3) Madagascar	511	346	158	93	68	0.9784 (0.0015)	0.0207 (0.0105)	1996–2001
**(X) Northern Indian Ocean**								
Oman	38	23	14	8	25	0.6913 (0.0520)	0.0179 (0.0094)	2001–2002
**Totals**	**1527**	**860**	**524**	**162**	-	-	-	

Region C1 groups samples from Mozambique (*M*) and Eastern South Africa (*ESA*), and Region C3 groups samples from Antongil Bay (*AB*) and Southern Madagascar (*SM*). Haplotype (h) and nucleotide (π) diversities, as well as their standard deviations are provided. Numbers of males and females do not always add up to the sample size, given that the dataset contains individuals sex. Duplicate samples were removed from the analysis.

Skin tissues were mostly obtained from biopsies [50] and to a lesser extent from sloughed skin, or stranded specimens (primarily Region X). Samples were preserved in 95% Ethanol or salt saturated 20% Dimethyl Sulfoxide solution (DMSO) and later stored at −20°C until processed. Additional information regarding samples is detailed in Table 1 and elsewhere [37], [51], [52], [53]. Total genomic DNA was extracted from the epidermal layer of biopsies using proteinase K digestion followed by a standard Phenol/Chloroform extraction method [54] or using a DNAeasy tissue kit (Qiagen).

A 520 bp fragment within the mtDNA control region [55], [56] was amplified using Polymerase Chain Reaction (PCR). PCR products were cycle-sequenced (both forward and reverse) with dye-labeled terminators following conditions recommended by the manufacturers. Sequence reactions were analyzed using an ABI-Prism model 377 DNA Sequencer, 3700 or 3730 Genetic Analyzer (Applied Biosystem®, Foster City, CA) or a MegaBACE 1000 DNA Sequencer (GE Healthcare). Duplicate samples identified based on genotype identity by Pomilla (2005), were removed from the final analysis. Sex-determination was accomplished by PCR amplification and subsequent *Taq I* digestion of homologous regions on the X and Y chromosomes (ZFX/ZFY) [57].

### Data analysis – Population Structure

DNA sequence variation patterns were characterized into mtDNA haplotype definitions for this species. From the 520 bp mtDNA Control Region fragment, a 486 bp consensus region that contains the majority of variable nucleotide positions in the mtDNA control region of humpback whales was examined for all samples [56]. Sequences for this portion of the mtDNA Control Region were aligned for each individual in *MacClade v. 4.01* [58] and *Sequencher v. 4.5* (Gene Codes Corp. Ann Arbor, MI).

The diversity of humpback whale mtDNA sequences was estimated at both the haplotype and nucleotide level [59] using *Arlequin 3.11* [60]. The geographic variation of haplotypes was quantified using the Analysis of Molecular Variance framework [61] as implemented in the software Arlequin 3.11 [60]. This procedure calculates standard variance components and an array of haplotypic correlation measures for population structure, referred to as Φ-statistics. The Φ_ST_ is analogous to Wright's F-statistic and to other genotype correlations used for the study of population structure [62], [63], [64]. The significance of the observed Φ or F-statistics was tested using the null distribution generated from 5,000 non-parametric random permutations of the data matrix variables. No correction for simultaneous tests was applied to significance levels of pairwise comparisons [65], [66]. A corresponding Chi-square analysis was conducted in DNnaSP [67].

Given the large degree of sampling for each region, we are able to investigate population structure at different spatial scales, as well as controlling for sex-biased structure. The data were first partitioned according to Breeding Stocks (A, B, C, and X) and then into sub-populations within Breeding Stocks (B1, B2, C1, C2, C3; see Figure 1 and Table 1) [33]. Data were further divided by gender to evaluate the effect of sex on population structure for all breeding stocks. For effects of sex, we evaluated differences between males, females, and males plus females, and whether the incorporation of non-sexed animals into the sample pool produced changes in our evaluation of sex effects on population structure.

### Data Analysis-Migration Rates between Breeding Grounds

In order to estimate effective migration rates and divergence times between neighbouring breeding stocks in a stepping-stone fashion (Stock A vs. Stock B, Stock B vs. Stock C, Stock C vs. Stock X) we used a maximum-likelihood framework based on coalescence theory, implemented in the programs MDIV and MIGRATE 2.0.3 [68], [69]. Our rationale was to use MDIV to provide estimates of symmetric migration rates and explore the possibility of asymmetric migration included in the MIGRATE algorithm. For the latter, we subdivided Region C1 into Mozambique (*M*) and Eastern South Africa (*ESA*), and C3 into Antongil Bay (*AB*) and Southern Madagascar (*SM*) in order to assess potential differences in gene flow directionality between these sub-populations.

MDIV was utilized to produce effective migration rate and divergence time estimates among neighbouring Breeding Stocks (A, B, C and X). This package uses a Markov Chain Monte Carlo (MCMC) procedure to jointly estimate multiple parameters for pairs of populations in a Maximum Likelihood framework. MDIV estimates the migration rate per gene per generation between populations scaled by the effective population size (M = 2*N_e_*m), the time since the two populations diverged scaled by the effective population size (T = t/2 *N_e_*), and the parameter theta (θ), which is a product of the effective population size and the mutation rate of the studied gene region, (θ = 4*N_e_*μ).

Markov chains of 5×10^6^ cycles were run with a burn-in time of 5×10^5^ cycles to minimize dependence on initial conditions. The maximum value for the theta parameter was theta = 0. The choice of theta = 0 provides the model with a flat (uninformative) prior hypothesis, which has the least influence on the parameter estimation. Values for Mmax (maximum value for the scaled migration rate) and Tmax (maximum value for the scaled divergence time) were the result of a sensitivity analysis to assess the Markov Chain convergence with different values for both parameters. A Tmax of 5 was well above the estimated T in all of our sensitivity computations, therefore proving an appropriate choice for the parameter estimation. The Mmax used for estimating M varied between Tmax = 10 to Tmax = 35 depending on the population comparison (Mmax A-B = 30, Mmax B-C = 35, Mmax C-X = 10). A minimum of ten converged runs for each comparison was used to provide average M values and corresponding standard deviations.

MIGRATE provides estimates of *M* (*m*/*μ*) and Θ (2*N_e_μ*) where *m* is the immigration rate, μ the mutation rate, and *N_e_* the effective population size. The product Θ*M* results in the number of immigrants per generation 2*N_e_m* (from now on reported simply as *N_e_m*). We adopted a migration matrix model allowing for asymmetric migration rates between regions and variable subpopulation sizes. The following Markov chain scheme was implemented: 15 short chains (dememorization: 10,000 genealogies, recorded genealogies: 500, sampling increment: 100), and one long chain (dememorization: 10,000 genealogies, recorded genealogies: 40,000, sampling increment: 100).

## Results

Table 1 illustrates the sample sizes for each sampling site within the wintering Breeding Stocks A, B, C, and X. A consensus region of 486 bp of the mtDNA control region was assembled in which a total of 162 maternal haplotypes (Genbank accession numbers GQ913691-GQ913852) was detected from 25–72 polymorphic sites (shown in Table 1 with numbers of haplotypes for each area). A subset of samples from Brazil (n≈20) had slightly fewer than 486 bp of sequences at the 3′ end of the Control Region [51]. However, incorporation of missing data did not affect the assignment of these sequences to haplotype definitions as other variable sites defined a particular haplotype. When polymorphic sites defining haplotypes in these samples were not available, and sequences matched to more than one haplotype, alternative scenarios of matching to other haplotypes were tested to assess their influence on the larger dataset. No significant differences were found. Haplotype diversity ranged from 0.974–0.980 for Breeding Stocks A, B, and C to the lowest haplotype diversity of 0.691 for Oman in Stock X. Nucleotide diversity estimates ranged between 0.018 and 0.025 for all four regions.

For the global AMOVA analysis, significant differences were found between the four Breeding Stocks A, B, C, and X for both **Φ**_ST_ and *F*_ST_ (Table 2). Chi-square tests were also highly significant (Table 3). However, nearly all of the molecular variance was attributed to differences in ‘within-site’ variation detected for both test statistics. Sex (males, females, and females+males [no unknowns]) had no significant effect on global population structure as the overall AMOVA results and Chi-square tests were highly significant for each gender stratum (Tables 2 and 3). However, males exhibit smaller degrees of genetic structure than females and males + females. Any plausible inter-annual comparisons were not significant (data not shown), indicating that there are no temporal sampling influences on the results.

**Table 2:** AMOVA results for breeding areas A, B, C and X of Southern Hemisphere humpback whales using molecular distances (Φst) and haplotype frequencies (Fst).

Samples	Stock	Global Fst	(p value)	Global Φst	(p value)
All samples	ABCX	**0.01849**	(0.00000)	**0.01131**	(0.00000)
M+F	ABCX	**0.02011**	(0.00000)	**0.01232**	(0.00000)
Females	ABCX	**0.02145**	(0.00000)	**0.01476**	(0.00099)
Males	ABCX	**0.01873**	(0.00000)	**0.01203**	(0.00089)

The AMOVAs (or ‘Global’ value) are shown for the entire dataset (All samples), animals of known sex from molecular sexing (M+F), females and males. The P-value is the probability of a more extreme variance component or F-value than that observed, in comparison to a null distribution of these values on 5,000 random permutations of the data matrix. Significant values (p<0.05) are highlighted in bold.

**Table 3:** Chi-Square test for differences in haplotype frequencies for four breeding Regions (A, B, C and X) of Southern Hemisphere humpback whales.

Samples	Stock	X^2^	p	DF
All samples	ABCX	1143.127	**0.000**	390
M+F	ABCX	967.074	**0.000**	375
Females	ABCX	524.924	**0.000**	300
Males	ABCX	616.676	**0.000**	330

All strata based on sex of animals are shown. The P-value is the probability of a more extreme variance component or F-value than that observed, in comparison to a null distribution of these values on 1,000 random permutations of the data matrix. Significant values (p<0.05) are highlighted in bold.

When either the total sample, males plus females or females alone were tested, all pairwise comparisons among Breeding Stocks and sub-populations within Breeding Stocks using *F*_ST_ were significantly different (p<0.05) from one another, except between C2 vs C3 (Tables 4, 5, 6). When only males were considered, all pairwise comparisons were significantly different with the exception of the following: B1 vs. B2 or C2; B2 vs. C1, C2 or C3; C1 vs. C2 or C3; and C2 vs. C3 (Table 7). For pairwise comparisons based on **Φ**_ST_, the following comparisons were statistically significant when using either all samples available or males+females: A vs. B1; B1 vs. C1or C3; and X vs. all population comparisons (Tables 4 and 5). For females: A vs. B1, B2 and C2; B1 vs. C1and C3; and X vs. all comparisons were statistically significant (Table 6). For males: A vs. B1, B2 and C3 were significantly different, while B1 vs. C3 was also significantly different, and all males from Stock X were statistically different from all other stocks (Table 7).

**Table 4:** Pairwise measures of genetic divergence in various populations of Southern Hemisphere humpback whales, using all samples (Table 4), males + females (Table 5), females only (Table 6) and males only (Table 7).

	A	B1	B2	C1	C2	C3	X
A		**0.0041**	0.0042	0.0037	0.0066	0.0036	**0.1034**
B1	**0.0073**		0.0000	**0.0049**	0.0016	**0.0043**	**0.0939**
B2	**0.0098**	**0.0030**		0.0019	0.0000	0.0030	**0.1057**
C1	**0.0148**	**0.0061**	**0.0057**		0.0019	0.0000	**0.0948**
C2	**0.0166**	**0.0084**	**0.0064**	**0.0052**		0.0002	**0.0963**
C3	**0.0120**	**0.0072**	**0.0053**	**0.0029**	0.0020		**0.0797**
X	**0.1473**	**0.1302**	**0.1280**	**0.1257**	**0.1175**	**0.1163**	

Pairwise Φst and Fst values are above and below the diagonal, respectively. Significant values are highlighted in bold.

**Table 5 pone-0007318-t005:** Pairwise measures of genetic divergence in various populations of Southern Hemisphere humpback whales, using all samples (Table 4), males + females (Table 5), females only (Table 6) and males only (Table 7).

	A	B1	B2	C1	C2	C3	X
A		**0.0118**	0.0106	0.0058	**0.0136**	0.0046	**0.0887**
B1	**0.0143**		0.0000	**0.0047**	0.0019	**0.0041**	**0.0899**
B2	**0.0168**	**0.0025**		0.0008	0.0000	0.0018	**0.1000**
C1	**0.0202**	**0.0061**	**0.0055**		0.0016	0.0000	**0.0891**
C2	**0.0250**	**0.0089**	**0.0071**	**0.0055**		0.0000	**0.0902**
C3	**0.0166**	**0.0072**	**0.0051**	**0.0030**	0.0021		**0.0767**
X	**0.1678**	**0.1299**	**0.1299**	**0.1246**	**0.1163**	**0.1160**	

Pairwise Φst and Fst values are above and below the diagonal, respectively. Significant values are highlighted in bold.

**Table 6 pone-0007318-t006:** Pairwise measures of genetic divergence in various populations of Southern Hemisphere humpback whales, using all samples (Table 4), males + females (Table 5), females only (Table 6) and males only (Table 7).

	A	B1	B2	C1	C2	C3	X
A		**0.0550**	**0.0299**	0.0210	**0.0378**	0.0301	**0.1427**
B1	**0.0237**		0.0035	**0.0116**	0.0034	**0.0056**	**0.0733**
B2	**0.0278**	**0.0016**		0.0000	0.0000	0.0000	**0.0827**
C1	**0.0426**	**0.0102**	**0.0105**		0.0000	0.0000	**0.0876**
C2	**0.0366**	**0.0102**	**0.0078**	**0.0073**		0.0000	**0.0676**
C3	**0.0258**	**0.0088**	**0.0064**	**0.0086**	0.0029		**0.0648**
X	**0.1696**	**0.1144**	**0.1093**	**0.1087**	**0.0861**	**0.0997**	

Pairwise Φst and Fst values are above and below the diagonal, respectively. Significant values are highlighted in bold.

**Table 7:** Pairwise measures of genetic divergence in various populations of Southern Hemisphere humpback whales, using all samples (Table 4), males + females (Table 5), females only (Table 6) and males only (Table 7).

	A	B1	B2	C1	C2	C3	X
A		**0.0359**	**0.0386**	0.0185	0.0193	**0.0298**	**0.0854**
B1	**0.0235**		0.0000	0.0053	0.0000	**0.0027**	**0.0811**
B2	**0.0225**	0.0015		0.0057	0.0000	0.0003	**0.0918**
C1	**0.0210**	**0.0056**	0.0032		0.0000	0.0000	**0.0740**
C2	**0.0281**	0.0002	0.0000	0.0000		0.0000	**0.0771**
C3	**0.0230**	**0.0060**	0.0033	0.0016	0.0000		**0.0669**
X	**0.1681**	**0.1256**	**0.1313**	**0.1215**	**0.1412**	**0.1111**	

Pairwise Φst and Fst values are above and below the diagonal, respectively. Significant values are highlighted in bold.

The highest effective migration rates resulting from our analysis utilizing MDIV were between Breeding Stocks B and C, followed by the estimated effective migration rate between Breeding Stocks A and B, with the lowest migration between Breeding Stocks C and X. Population divergence time estimates are inversely related to the effective migration rates, with divergence time between Breeding Stocks C and X being the greatest (Table 8). Using MIGRATE to assess asymmetric effective migration patterns, the highest degrees of migrants inferred occurs from: Breeding Stock B1 to A; C2 to C1-*ESA*; C3 to B2, C2 or C1-*ESA*; and C1-*ESA* to B2 (Table 9). Rates of migration with *Nem*/generation ≤5, but >1, suggests limited gene flow from B1 to B2 or C3; C1-*M* to B1 or B2; C1-*ESA* to B1 or C3. With ≤1 *Nem*/generation based on mtDNA lineages analysis, the following comparisons are characterized by either a very low or a lack of gene flow: Breeding Stock B2 to B1, C3-*SM*, C2, C1-*M* or C1-*ESA*; C to X; and X to C. Interestingly, limited or very low gene flow rates were detected for whales between the two sub- populations in Breeding Stock B (B1 and B2), compared to high gene flow rates for whales within Breeding Stock C.

**Table 8:** Likelihood-based estimates of migration rate and divergence time between population pairs of Southern Hemisphere humpback whales, performed with software MDIV.

	M [2Nem] (SE)	T [t/2Ne] (SE)	θ [4Nμ] (SE)
A-B	25.935 (1.612)	0.063 (0.017)	15.811 (0.458)
B-C	32.547 (1.12)	0.071 (0.01)	17.593 (0.155)
C-X	5.179 (0.335)	0.145 (0.022)	17.131 (0.354)

At least ten runs were averaged to obtain the shown values. M and T represent the population migration rate and population divergence time per generation scaled by population size, respectively, and theta is directly proportional to the effective population size and the mutation rate, according to θ = 4Nμ.

**Table 9:** Estimated number of migrants per generation (Nem) exchanged between neighbouring Southern Hemisphere humpback whale Breeding Regions, as estimated using the program MIGRATE.

	A+	B1+	B2+	C3N+	C3S+	C2+	C1N+	C1S+	X+
A	-	29.555	2.454	*	*	*	*	*	*
B1	3.492	-	0.386	0.193	0.394	2.125	2.099	1.159	*
B2	1.551	2.183	-	37.132	10.854	2.326	1.551	14.240	*
C3N	*	2.123	5.601	-	1.600	2.836	6.531	1.067	0.267
C3S	*	4.885	0.696	6.613	-	0.348	0.348	2.436	1.139
C2	*	13.054	0.000	27.308	4.656	-	0.665	0.665	0.693
C1N	*	20.749	0.000	19.680	5.238	2.829	-	0.655	0.000
C1S	*	25.579	0.000	1.518	1.012	1.012	0.506	-	0.506
X	*	*	*	0.285	0.570	0.285	0.285	0.285	-

Magnitude and directionality are shown by reading the first row (ie A+) in the first place, and then matching the appropriate cell with regions listed in leftmost column. (*) Comparisons between Breeding Regions A or B vs. X, and A vs. C are omitted, as they are not neighbouring Breeding Stocks. Sub populations in Breeding Regions C1 and C3 were used for the MIGRATE analysis.

## Discussion

### Oceanic Population Structure

Overall, the tests for population differentiation based on haplotype frequencies and molecular distances were significant among Breeding Stocks A, B, C and X, as well as between particular sub- populations within these Stocks (only the values for males alone showed less discrimination, and these will be discussed below). Therefore, the AMOVA, Chi square and pairwise test results presented here generally support the current IWC designation of Breeding Stocks A, B, C, and X. The magnitude of these fixation indices is consistent with other comparisons of SH humpback whale breeding stocks [49]. On an evolutionary scale, these data suggest long-term maternal transmission of migration routes and fidelity to breeding grounds for whales in the South Atlantic and Southwestern Indian Ocean that reinforce population structure, with a degree of gene flow that likely erodes strong differentiation.

Within the Southern Atlantic Ocean, humpback whales congregate in the waters off northeastern Brazil (Abrolhos Banks, Breeding Stock A) and throughout the Gulf of Guinea (Breeding Stock B). Our findings indicate differentiated demographic aggregations of breeding individuals in these two stocks with small evolutionary divergence, as evidenced by significant differences in haplotype frequencies, coupled with small and non-significant molecular distances and high migration rates. Recent genotypic capture-recapture, satellite telemetry studies, and a limited number of photographic comparisons have established provisional migratory connections between breeding grounds and feeding areas for each of these two populations based on movements of a number of individuals [51], [70], [71].

Using the available evidence collectively, it appears the majority of whales in Breeding Stock A retain their fidelity to breeding grounds off Brazil and to feeding areas in the western South Atlantic, where historical concentrations once existed [51], [71]. Similarly, some humpback whales that breed in the Gulf of Guinea feed in areas south-west of Southern Africa in the eastern South Atlantic near Bouvet Island (54°S) [72], classified by IUCN as a maritime Antarctic Island, south of the Antarctic Polar Front [72], [73]. While no transoceanic migration events have been detected, our data shows evidence of exchange or mixing between these two populations on opposites sides of the Atlantic Ocean. Interestingly, an acoustic analysis of humpback whale song in a single breeding season found identical song structure amongst the whales of Breeding Stocks A and B [74]. The nature of cultural transmission of song [75], [76] and change in theme composition that occurs through time, coupled with significant, but low genetic differentiation between these stocks, support contact among males of these two populations somewhere in their annual migratory cycle or on their feeding areas [36]. Mediation of gene flow may occur south of the breeding grounds or on feeding areas. Singing can occur on the feeding areas [77] where mixing between whales from different populations may be most likely to occur.

The differentiation between whales from Breeding Stocks B and C is largely evident through statistically significant partitioning between *F_ST_* or chi-square values. When assessing the degree of differentiation in relation to migration patterns, the effective numbers of migrants from these populations (Tables 8 and 9) are generally the highest for whales in two different oceanic regions. Direct movements of an individual whale based on genetic capture-recapture have also been detected between these regions [78]. The exact path for oceanic inter-change remains unknown, and the level to which it recurs is not understood. The potential for this degree of gene flow to be mediated through mixing of whales on common feeding areas in the Southern Oceans (south of 50 degrees), and resultant movements to neighbouring Breeding Stocks where gene flow would occur, is being investigated using mtDNA and microsatellites (Loo et al. in prep). Population genetic theory indicates that *F_ST_* values of 0.01 correspond to approximately 25 migrant individuals per generation (which are consistent with results from MIGRATE). For humpback whales, this could amount to 1 or 2 migrants per annum, and would suggest these populations are demographically and temporally structured on the Breeding Grounds, yet can and do readily exchange individuals [78].

With respect to the lack of differentiation among pairwise comparisons using molecular distances between the South Atlantic and Southwestern Indian Ocean (Breeding Stocks B and C), it is possible that the lack of structure is due to retained common ancestry. Phyleogeographic analyses of these mtDNA lineages also support a high degree of ancestry [data not shown, but also see 51]. All of the maternal lineages identified in this region represent evolutionary closely related groups, defined by low molecular distances between haplotypes. However, it is difficult to differentiate between the retention of ancestral polymorphism and levels of recent connectivity. Given the estimated migration rates between B2 and C1, and the higher statistical significance of the haplotypic distances as compared to molecular distances, we would favor the scenario of some degree of contemporary exchange, for example, between these two adjacent sub- populations. This scenario as determined from the mtDNA population structure and gene flow analyses is consistent with the inter-ocean migration event reported in [78].

High statistical significance for both haplotype frequencies and molecular distances indicates strong genetic structure between stock X and all other Breeding Stocks including Stock C. To date, no photographic matches have been found between Stocks X and C [79]. The majority of the population structure results are highly significant (*F_ST_* ∼0.1, Phi-ST ∼0.9, and all significance levels ≤0.05), more so than any other pairwise comparison between Breeding Stocks in this study and elsewhere in the SH [49]. The *F_ST_* values detected between Breeding Stocks C and X (*F_ST_* ∼0.11–0.17) are among the highest recorded for population differentiation among any humpback whale populations worldwide, and begin to approach values representing long-term phylogeographic patterns (*F_ST_* ∼0.5) of other marine species, such as marine turtles [14].

Furthermore, the effective number of migrants per generation between Breeding Stock C sub- populations and Region X is the lowest when contrasted with any other pairwise comparisons in this study. These results taken collectively with the evidence that all but one of the haplotypes are shared between Northern Indian Ocean and Southwestern Indian Ocean, suggest that a high degree of shared ancestral polymorphism in mtDNA control region lineages is maintained between these highly differentiated populations in the Southwestern and Northern Indian Oceans. These results are consistent with an isolated population of humpback whales in the northern Indian Ocean.

Research indicates that the population size off the coast of Oman is small (<200 individuals) [53]. However, other portions of the expected range of the population in the Northern Indian Ocean have not been systematically surveyed [53]. This could potentially influence interpretations concerning population structure. The high haplotype diversities among the other populations in this study, coupled with a smaller population size from Breeding Stock X, could create a bias toward statistically significant differentiation when contrasted with populations with larger sample sizes. This could occur if whaling had a differential impact on particular maternal lineages (for example, more common lineages or lineages associated with particular geographic areas). Although in the case with humpback whales from Breeding Stock X we do find private mtDNA haplotypes, the majority of lineages are the same as those found among the other Breeding Stocks, differing significantly in frequency. Foetal evidence from whaling data indicates that northern Indian Ocean humpback whales adhere to a NH humpback breeding cycle with mating and calving occurring between January and May [35], providing a likely barrier to inter-breeding with whales in the SH [53]. Further evidence of feeding and breeding behaviours (recording of song), as well as the year-round sightings of individual whales, suggested that whales in this area may not exhibit typical migratory characteristics associated with NH or SH populations. Given the high degree of population differentiation inferred from mtDNA and the low population size, the survival of this small and isolated population is of great concern to the international scientific and conservation community [53].

### Sub-Population Structure

Nearly all B and C sub- population comparisons (with the exception of C2 vs. C3) were statistically significant when assessed by the *F*_ST_ index for all samples, males plus females, and females alone (Tables 4, 5 and 6). The lack of significant *F*_ST_ for C2 and C3 is consistent with photographic recaptures between these sub- populations [33]. Samples and sampling effort from whales in the Mozambique Channel (C2) have been limited. A more complete analysis of whales in the C2 sub-population is needed to better evaluate the degree of connectivity of the C2 and C3 sub- populations. Comparisons between C1 (East South Africa) and C3 (Antongil Bay) showed significant *F*_ST_ values (albeit low) and high rates of gene flow. These results, taken together with the population structure data, could suggest current demographic independence, with some degree of contemporary exchange, and likely a high degree of shared ancestry among whales in sub-populations of Breeding Stock C in the Southwestern Indian Ocean. However, further analyses that critically examine interchange of individuals, directionality, and whether these represent mature adults are needed for discriminating between ecological, reproductive and evolutionary influences in population structuring.

Conversely, whales sampled from Breeding Stock B1 are significantly differentiated from whales of Breeding Stock B2, and very low gene flow rates are detected. This is contrary to the most fundamental expectations of isolation-by-distance, where whales from the adjacent B1 and B2 regions were thought to be part of the same breeding population. Satellite telemetry studies show two individuals moving from B1 to areas well to the west of survey/sampling range of B2, offshore from the Walvis Ridge [72]. The mtDNA differentiation of B1 and B2 sub-populations presents a very interesting case in the field of molecular ecology where populations appear to be differentiated in the marine environment in the absence of a geographic barrier, possibly due to environmental or ecological factors, or some undetected temporal stratification of matrilineal lineages within the sampling of whales from the B2 sub- population.

Recent surveys indicate that humpback whales are found in the southern range of B2 (west South Africa) during summer [80], and photographic identification shows individuals occurring in these waters in several different months. Combined with historical catches off Namibia during austral summer months [81], recent records of whales defecating and feeding off the west coast of South Africa, and the known high productivity of the southern Benguela region [82], these data suggest that the southern section of the B2 sub- population may serve more as a feeding location and migratory corridor and that a second unsurveyed breeding population may exist further north in B2 [33]. These animals would likely breed off Angola, which had known concentrations for 19^th^ and 20^th^ century whalers [83] or potentially as far south as Southern Angola near the Agulhas-Benguela Front. Animals sighted and caught during coastal whaling operations off Namibia (Luderitz and Walvis Bay) are likely to be part of the population undertaking seasonal migration from winter breeding grounds further north. However, further surveying and sampling is necessary to confirm relationships to B1 and B2 sub-populations.

### Influence of sex-biased movements and sampling

Evaluating mtDNA control region lineages stratified by sex provides a preliminary evaluation of sex-biased effects of dispersal in terms of population structure, although a comparative analysis with bi-parentally inherited markers would be necessary to fully account for male-driven population structure. Because females pass on their mtDNA haplotype to their offspring, they can homogenize the mtDNA structure through dispersal coupled with reproductive events in different populations. Males, on the other hand, do not significantly influence mtDNA structure. If despite this fact females show higher mtDNA structure than males, it would only be conservative to suggest that dispersal is biased toward males and that females exhibit a significant degree of phylopatry. The typical presumption in the social system of baleen whales is that despite the fidelity to particular areas, males will generally disperse more widely than females, resulting in higher levels of female mtDNA structure as compared to male mtDNA structure. When comparing our male and female fixation indices we observe a markedly higher female structure (i.e. a change in detecting a significance level for population pairwise comparisons) for F*_ST_* and **Φ**_ST_ pairwise comparisons between Stocks A, B, C and X (Tables 2 and 4–7). Furthermore males exhibit lower degrees of genetic structure when contrasted with all other types of data-groupings for F*_ST_* comparisons, and varying degrees of genetic structure for **Φ**_ST_ pairwise comparisons. Whereas these data would support the assumption of female phylopatry and male dispersal for humpback whales in our study area, we emphasize that mtDNA results should be cautiously evaluated in the absence of nuclear or male-specific molecular markers.

At a smaller spatial scale, most pairwise F*_ST_* and **Φ**_ST_ comparisons among males between Breeding Stocks B and C were not significant (Table 7). The mtDNA AMOVA and pairwise F*_ST_* are again suggestive of some degree of female fidelity to Breeding Stocks, and would be more consistent with patterns of increased male dispersal and migration between sub- populations within Breeding Stocks or between Breeding Stocks.

For **Φ**_ST_ tests, females generally exhibited non-significance in the pairwise comparisons for many of the sub- population comparisons. Although these results are largely incongruent with the *F_ST_* pairwise comparisons, this is an expected outcome when stocks present small evolutionary divergence. In addition, these results may be biased by the fact that, with the exception of Brazil (males = 20;females = 18), West South Africa (55∶49) and Mayotte/Comoros (57∶18), there is a considerable sampling bias toward males on these breeding grounds [84] (see Table 1). This is a typical problem on humpback whale breeding grounds, and it is unclear whether these are true “sampling” biases (i.e. easier acquisition of samples from males) or whether females are truly not available (i.e. sex bias). With relatively fewer known females available for sampling, the most common haplotypes increase in frequency, typically missing rare or infrequent haplotypes in sampling events. This results in significantly lower molecular distances among the available haplotypes, contributing to non-significant **Φ**_ST_ values, but still allowing for significant differences between haplotype frequencies *per se*, and therefore significant *F_ST_* values. This is one of the more parsimonious explanations for the observed pattern given the genetic results and issue of female availability on the breeding grounds.

### Biogeography and Migration Patterns

Interesting and consistent biogeographic patterns for marine species are beginning to emerge for migratory marine species in their environments. From detailed studies of mtDNA control region sequences of green sea turtle populations (*Chelonia mydas*) in the Southwestern Indian Ocean, Bourjea et al. [15] challenged traditional hypotheses concerning the movements of green sea turtles between the Atlantic and Indian Oceans. While colder waters of the Benguela Current System have traditionally been considered a thermal barrier to gene flow for some warm-water marine species, Bourjea et al. [15] found evidence that movement of sea turtles between these ocean basins has occurred, although not at high rates. Such inter-ocean basin movement may be facilitated by Agulhas Current eddies or rings which convey significant quantities of Agulhas Current water into the South Atlantic. This oceanographic discontinuity would affect a stenothermic species such as a marine turtle, but would likely not have as much relevance to humpback whales during migration. However, Perrin [85] has suggested that the interface between Agulhas and Benguela currents has likely had an effect for movements of cetaceans over evolutionary time. Furthermore, recent research is beginning to reveal that water temperature can play an important role for humpback whale breeding grounds [86].

The only movement directly detected for humpback whales between these ocean basins is from East to West, and represent movements from the Indian to the Atlantic Ocean [78]. The *Nem* values as calculated in MDIV and MIGRATE, being larger from Stock C to Stock B2 than from B2 to C, support this finding. Prevalent migration from the Indian to the South Atlantic Ocean via southern Africa has been proposed for the big eye tuna (*Thunnus obesus*), whereas higher migration in the opposite direction has been suggested for hammered sharks and (*Sphyrna lewini*) and the green turtle (*Chelonia mydas*) [15], [87], [88]. Our data on humpback whales can be explained either by exchange events between both Breeding Stocks around the South African coast or by movement events in the Southern Ocean during the feeding season, followed by switching of migratory routes. Whereas the first possibility would favour a relationship between humpback whale movement patterns and oceanographic features in the Southern African region, such local oceanographic features would have no influence over exchange events occurring in the feeding areas.

The location of the Angola Current/Benguela Current Front coincides with a major hiatus in the availability of genetic samples, most being either from Gabon (north of 5^o^S) or west south Africa (south of 32^o^S). Although a role for this Front as the possible divisor between the two Breeding Stocks along the West Coast of Africa [33], [36] is supported by mtDNA differentiation and satellite tagging data [72], this may be coincidental with the geographic nature of collected samples and requires further analysis particularly from humpback whales off the coast of Angola. Furthermore, from a biogeography perspective, the migratory destinations for breeding grounds in the eastern South Atlantic Ocean (Breeding Stock B1, near the equator) and in the western Indian Ocean (Breeding Stock C, considerably south of the equator, but within the tropics) raises the question about particular characteristics that humpback whales seek for a breeding ground. In this case, it may be that differences in water temperatures (≈24°C) help dictate movements to breeding grounds at oceanic scale [86]. Two SH humpback whale breeding grounds, one in the eastern equatorial Pacific (G Breeding Stock & central American Breeding grounds) and the other in the eastern South Atlantic/Gulf of Guinea (B1 Breeding Stock), occur north of the equator where SSTs ≥24°C. Examination of the location of breeding grounds for SH humpback whales indicates these two regions are considerably to the north of other SH breeding grounds [89], and suggest that the Benguela along with the Humbolt/Peru Current systems influence the location of breeding grounds for these populations [86].

### Summary and Management Implications

Most populations of humpback whales became severely depleted following intensive periods of whaling that included 18^th^ and 19^th^ century pelagic whaling [83], coastal whaling operations [90], and more mechanized commercial whaling and floating factories in the 20^th^ century [90], [91]. With the cessation on whaling of SH humpback whales in 1963 (and despite the continuation of illegal Soviet whaling activities into the early 1970's), certain depleted populations are now undergoing significant recoveries [89], [90], [92], [93], [94], [95]. Genetic assessments to evaluate population structure are the backbone of Management Unit (MU) designations, which have proven key elements of prioritization in conservation and management strategies [1].

The combined genetic evidence suggests that there is a significant degree of population structure for humpback whale Breeding Stocks within the Indian and South Atlantic Oceans. The Breeding Stocks in the Northern and Southwestern Indian Ocean are the most differentiated from each other, providing further evidence for the isolated nature of the population in the Northern Indian Ocean. Similar patterns of oceanic population structure are broadly observed in the Southeastern Indian Ocean, for Breeding Stock D off Western Australia, and in the South Pacific for Breeding Stocks E–G [49]. Based on an analysis of 1112 samples (not individuals) using mtDNA control region sequences, Olavarria et al. [49] found significant differentiation at the haplotype and nucleotide levels between 6 different breeding grounds, which fit within 4 larger Breeding Stocks, defined by the IWC. In that study, the authors suggest that their large-scale mtDNA comparisons demonstrate sufficient differentiation (F*_ST _*∼0.01–0.07) to support the defined Breeding Stocks, and sub-population structure in the South Pacific Ocean.

While significant mtDNA population structure clearly exists between Southeastern Atlantic and Southwestern Indian Ocean, (Stocks B and C), the magnitude of such structure is relatively weak in relation to the other comparisons in our study. This is also evidenced by the estimated migration rates, which suggest that some level of mixing occurs between humpback whales from these populations. The mtDNA results presented here show 1) significant genetic differentiation between oceanic Breeding Stocks 2) genetic differentiation between sub-populations with some degrees of gene flow between sub-populations within oceanic population structure, and 3) oceanographic phenomena likely influencing humpback whale population structure. An integrative approach combining our genetic analyses with bi-parentally inherited molecular markers and contemporary movement data in various population-modeling scenarios may provide a more precise framework for evaluating whale recovery from previous exploitation, and hence its resilience to current and emerging threats, including the possibility of resumed hunting.
